# Supplementary material for: Association between oral microbiome and five types of respiratory infections: a two-sample Mendelian randomization study in east Asian population
Source: Front Microbiol. 2024 Apr 10;15:1392473. doi: 10.3389/fmicb.2024.1392473 (PMC11039966; doi:10.3389/fmicb.2024.1392473)
Supplement: Supplementary file 1 [file Data_Sheet_1.zip › Supplementary Materials/MR plots of saliva/Tonsillitis/s__Streptococcus_pseudopneumoniae_G_mgs_1360/forest.pdf]

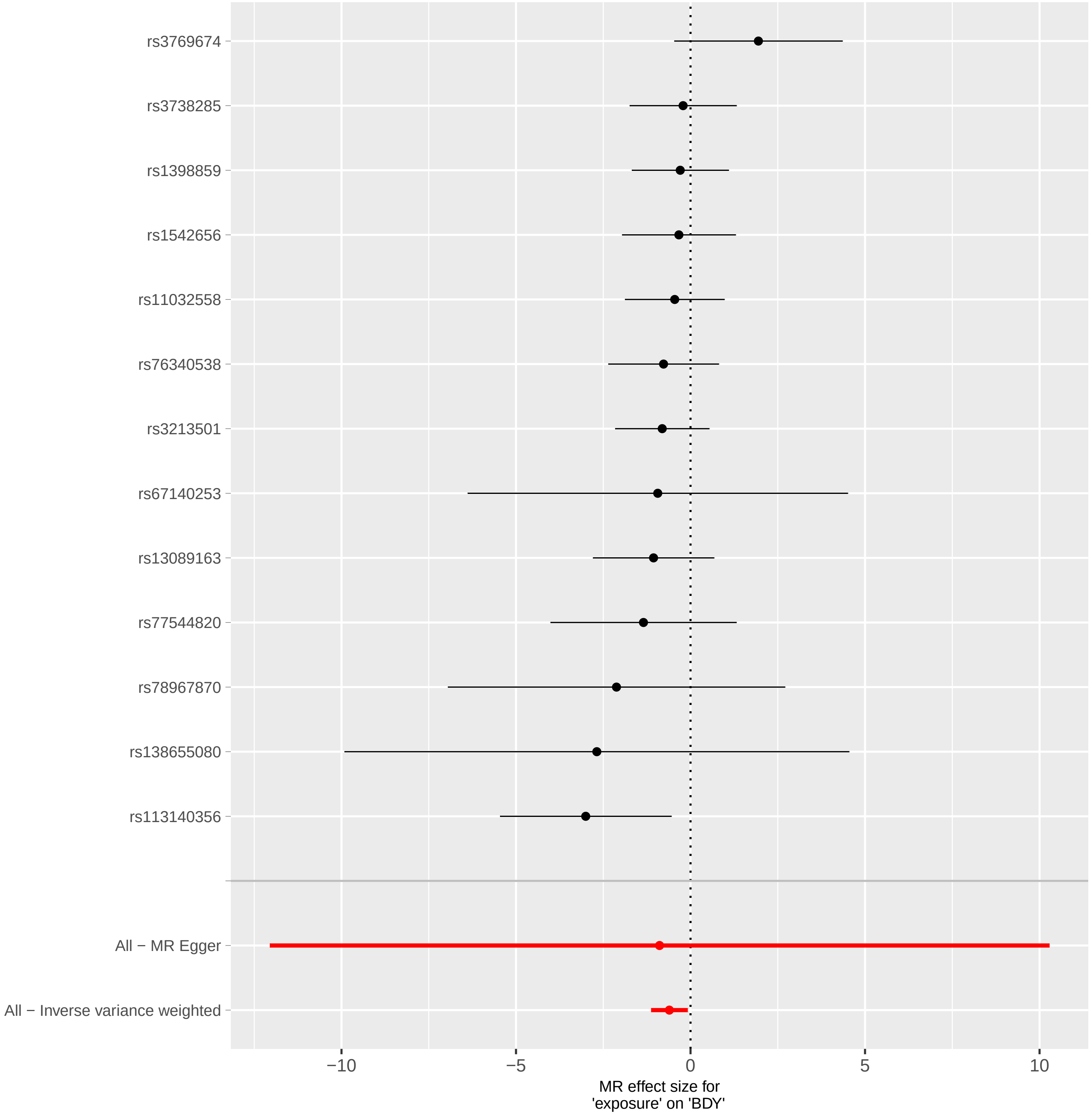

rs3769674
rs3738285
rs1398859
rs1542656
rs11032558
rs76340538
rs3213501
rs67140253
rs13089163
rs77544820
rs78967870
rs138655080
rs113140356
All − MR Egger
All − Inverse variance weighted
−10
−5
0
5
10
MR effect size for
'exposure' on 'BDY'
